# Deubiquitinating enzyme Usp12 regulates the interaction between the androgen receptor and the Akt pathway

**DOI:** 10.18632/oncotarget.2162

**Published:** 2014-07-08

**Authors:** Urszula L. McClurg, Emma E. Summerscales, Victoria J. Harle, Luke Gaughan, Craig N. Robson

**Affiliations:** Solid Tumour Target Discovery Laboratory, Newcastle Cancer Centre, Northern Institute for Cancer Research, Medical School, Newcastle University, Newcastle upon Tyne NE2 4HH, UK

**Keywords:** Androgen Receptor, Usp12, prostate cancer, Uaf-1, WDR20, PHLPP, PHLPPL, Akt

## Abstract

The androgen receptor (AR) is a transcription factor involved in prostate cell growth, homeostasis and transformation regulated by post-translational modifications, including ubiquitination. We have recently reported that AR is deubiquitinated and stabilised by Usp12 resulting in increased transcriptional activity. In this study we have investigated the relationship between Usp12, PHLPP and PHLPPL tumour suppressors in the regulation of AR transcriptional activity in prostate cancer (PC). PHLPP and PHLPPL are pro-apoptotic phosphatases that dephosphorylate and subsequently deactivate Akt. Phosphorylated Akt is reported to deactivate AR in PC by phosphorylation at Ser213 and Ser791 leading to ligand dissociation and AR degradation. In contrast, PHLPP- and PHLPPL-mediated dephosphorylation and inactivation of Akt elevates the levels of active AR. In this report we demonstrate that Usp12, in complex with Uaf-1 and WDR20, directly deubiquitinates and stabilises the Akt phosphatases PHLPP and PHLPPL resulting in decreased levels of active pAkt. Decreased pAkt in turn down-regulates AR Ser213 phosphorylation resulting in enhanced receptor stability and transcriptional activity. Additionally, we observe that depleting Usp12 sensitises PC cells to therapies aimed at Akt inhibition irrespectively of their sensitivity to androgen ablation therapy. We propose that Usp12 inhibition could offer a therapeutic alternative for castration resistant prostate cancer.

## INTRODUCTION

Prostate cancer (PC) is the most common non-cutaneous cancer affecting Western males, accounting for over a million cases diagnosed worldwide in 2012 according to WHO. Androgen receptor (AR) signalling plays a major role in prostate cancer development. The AR is a transcription factor that belongs to the nuclear hormone receptor family and regulates the expression of genes involved in prostate proliferation and apoptosis [1]. Deregulated AR activity disrupts the balance between proliferation and apoptosis leading to cellular transformation; and hence the receptor signalling cascade remains the primary therapeutic target for PC treatment [2]. After a preliminary stage of hormone-sensitive disease that is treated by androgen-deprivation therapy and agents directly inactivating the AR, patients progress to castrate resistant prostate cancer (CRPC) for which there is no curative treatment available with an extremely poor prognosis. Importantly, the AR remains a crucial driver of CRPC, as evidenced by elevated levels of the AR-regulated gene *PSA* and *AR* gene amplification and mutations, thus new strategies targeting the AR signalling cascade, both directly and indirectly, are likely to be efficacious in this disease state [3].

AR can be post-translationally modified by multiple proteins and those modifications affect its activity and stability. We and others have shown that the AR is ubiquitinated by a number of E3 ubiquitin ligases, including MDM2 [4], [5], CHIP [6], [7], RNF6 [8] and NEDD4 [9], [10] which results in proteosomal degradation and changes to transcriptional activity. However, very little is known about reversal of this modification in AR regulation. Usp26 was reported to deubiquitinate AR resulting in receptor deactivation or MDM2 ubiquitination followed by AR degradation depending on cellular context [11]. Additionally, Usp10 was shown to bind AR causing an increase in its transcriptional activity [12]. We have recently reported that Usp12, in complex with Uaf-1 and WDR20, can directly bind and deubiquitinate the AR resulting in increased receptor stability and transcriptional activity [13]. As a result, depleting Usp12 decreased PC cellular proliferation and increased cellular apoptosis suggesting it may be a potential target for CRPC therapy [13].

In this study we focused on the relationship between the AR and Akt pathways. The Akt pathway plays a pro-survival and pro-proliferative role and is involved in prostate carcinogenesis [14], [15]. The PI3K/Akt pathway and AR are reported to act within a feedback loop in prostate cells in which Akt directly phosphorylates AR at S213 and S791, resulting in promotion of receptor degradation by driving MDM2-mediated ubiquitination of the AR [16], [17], [5], [18]. Additionally, AR S213 phosphorylation represses the interaction between the AR and its cofactors ARA70, ARA54 and TIF-2 resulting in decreased transcriptional activity of the AR [16]. Similar effects were observed for PIM-1S phosphorylation of the AR at S213 [19]. In this process, S213, and not S791, was reported to be the primary site mediating the AR inhibition by Akt [16]. Importantly, clinical studies have demonstrated that S213 phosphorylation of AR correlated with pAkt and predicted decreased patient survival [20], [19].

The effects of Akt on AR can be opposed by the Akt phosphatases Pleckstrin Homology domain leucine reach repeat protein phosphatases, PHLPP and PHLPPL [21]. PHLPP dephosphorylates Akt2 and Akt3 and PHLPPL dephosphorylates Akt1 and Akt3 at S473 [21]. PHLPP and PHLPPL are reported to be lost in 30% and 50% of PC, respectively, highlighting their clinical importance [22]. PHLPP protein is regulated by ubiquitination by the SCF-b-TrcP complex leading to its proteosomal degradation [23]. Interestingly, this can be reversed by two closely-related Usp12 family members, Usp46 in colon cancer [24] and Usp1 in lung cancer [25] which both deubiquitinate and stabilise PHLPP. Additionally, Usp12 was predicted to interact with both of these phosphatases and a more recent report confirmed the interaction between Usp12 and PHLPP in colorectal cells [26].

Here we report that Usp12, in complex with Uaf-1 and WDR20, interacts with PHLPP and PHLPPL in PC cells resulting in their deubiquitination and protein stabilisation. Consequently, Usp12 decreases the levels of active phospho-Akt (pAkt). As a result Usp12 regulates the cross-talk between the Akt and AR pathways. As such, overexpressing Usp12 inhibits AR S213 phosphorylation resulting in increased AR stability and transcriptional activity. Additionally, depleting Usp12 sensitises PC cells to Akt inhibition irrespectively of their AR status. Therefore, we have deciphered a novel regulatory pathway of the AR that may be translationally-relevant in CRPC that is characterised by elevated pAkt.

## RESULTS

### The Usp12 complex interacts with PHLPP and PHLPPL

**Figure 1:**
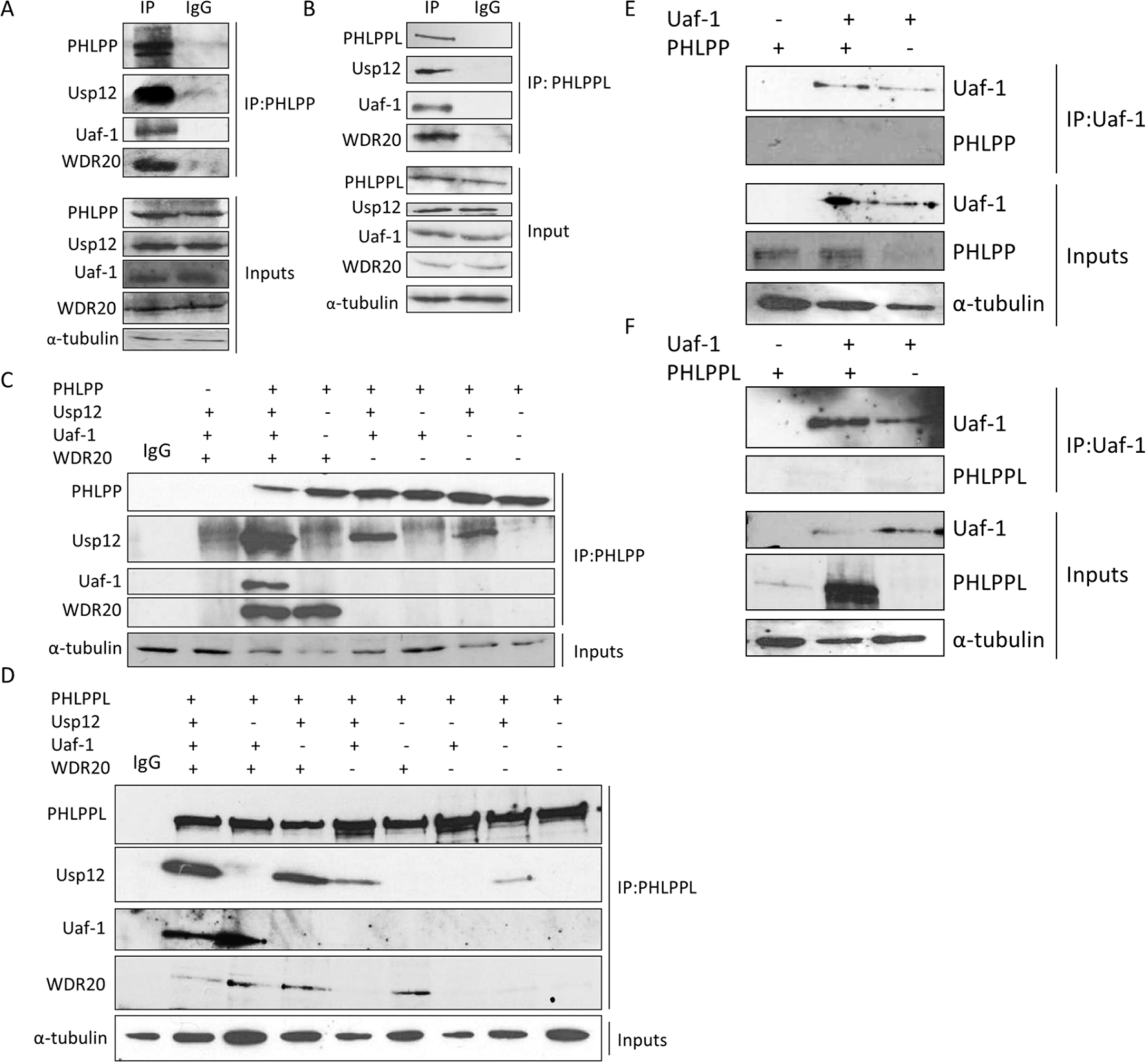
Usp12 in complex with Uaf-1 and WDR20 interacts with PHLPP and PHLPPL **(A-B)** LNCaP cells were harvested and lysates immunoprecipitated for either endogenous PHLPP **(A)**, PHLPPL **(B)** or non-specific IgG. **(C-D)** COS-7 cells were transfected with plasmids as indicated. 72h post transfection cells were harvested and lysates were immunoprecipitated (IP) with PHLPP **(C)** or PHLPPL **(D)** antibody followed by immunoblotting. **(E-F)** COS-7 cells were transfected with plasmids as indicated. 72h post transfection cells were harvested and lysates were immunoprecipitated (IP) with Flag antibody for Uaf-1-Flag followed by immunoblotting.

A previous proteomics study predicted PHLPP and PHLPPL as potential interacting partners of Usp12 [27]. To establish if this was the case in PC cell lines, we firstly investigated potential Usp12-PHLPP/L interactions in androgen-dependent LNCaP cells by immunoprecipitation. In agreement with the previous study, we found that both PHLPP (Figure 1A) and PHLPPL (Figure 1B) interacted with Usp12. Additionally, we also show that two components of the Usp12 complex Uaf-1 and WDR20 [28], [29] interacted with both phosphatase enzymes (Figure 1A/B). To assess the stoichiometry of the complex, we overexpressed PHLPP and PHLPPL together with the components of the Usp12 multimer and assessed by immunoprecipitation the minimal complex structure required for the interaction. Both Usp12 and WDR20 alone were able to interact with both PHLPP (Figure 1C) and PHLPPL (Figure 1D). Interaction between Uaf-1 and PHLPP or PHLPPL appears to be indirect, as in both cases, WDR20 was required for this association (Figure 1C-D). This is in agreement with previous reports demonstrating that WDR20 is the bridging component in the Usp12 complex required for the appropriate display of Uaf-1 and Usp12. We further confirmed the lack of direct interaction between Uaf-1 and PHLPP or PHLPPL using immunoprecipitation in the reverse direction (Figure 1E-F). It is possible that binding of PHLPP or PHLPPL to Uaf-1 requires a conformational change that is facilitated by Uaf-1 becoming part of the WDR20 and Usp12 complex.

### Usp12 deubiquitinates and stabilises PHLPP and PHLPPL and controls Akt activation status

**Figure 2:**
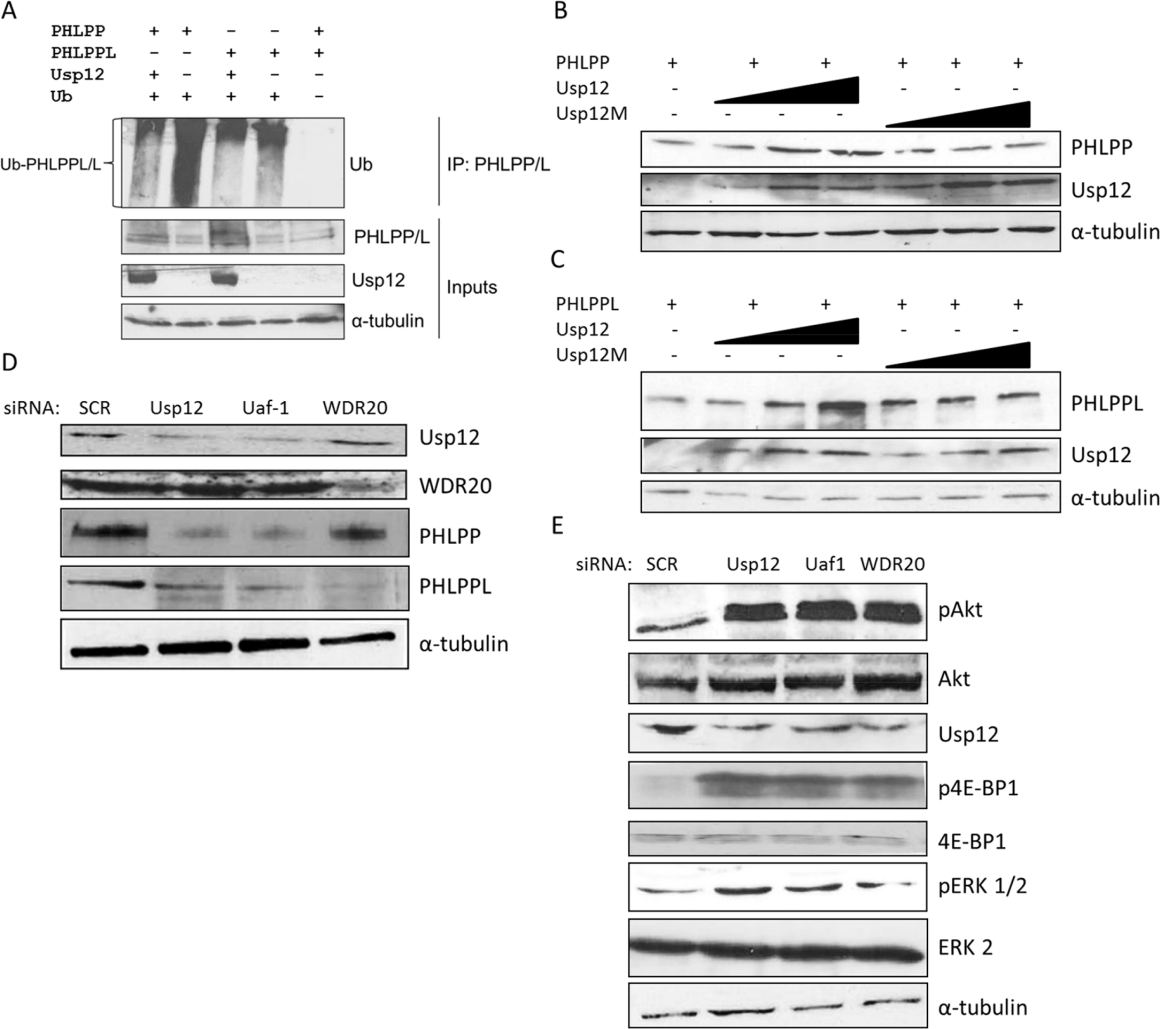
Usp12 deubiquitinates and stabilises PHLPP and PHLPPL and as a result controls the levels of pAkt **(A)** COS-7 cells were transfected with p-HA-PHLPP, p-HA-PHLPPL, p-His-Ub and p-Flag-Usp12 plasmids as indicated. 72h post transfection cells were treated with MG-132 and harvested 16h later. Lysates were denatured and subsequently immunoprecipitated (IP) with HA antibody (PHLPP and PHLPPL) followed by immunoblotting. **(B-C)** COS-7 cells were transfected with PHLPP **(B)** and PHLPPL **(C)** and with increasing amounts (75-300ng) of WT or C48A mutant Usp12 for 72h. Reactions were balanced with empty pCMV vector. Cells were lysed followed by immunoblotting. **(D-E)** LNCaP cells were treated with siRNA as indicated, at 96h cells were lysed followed by immunoblotting.

PHLPP protein stability was previously shown to be regulated by ubiquitination by SCF-b-TrcP complex [23], similar regulation probably occurs for PHLPPL. As Usp12 is a deubiquitinating enzyme we hypothesised that PHLPP and PHLPPL could be potential targets for ubiquitination reversal by Usp12. To assess this, we overexpressed ubiquitin, Usp12 and either PHLPP or PHLPPL in COS-7 cells prior to treatment with the proteosomal inhibitor MG-132, to maximise the levels of ubiquitinated phosphatase enzymes, followed by lysis under denaturing conditions that permits exclusive detection of ubiquitinated PHLPP or PHLPPL without contamination by interacting proteins. Lysates, were immunoprecipitated with anti-PHLPP or PHLPPL antibodies and the levels of ubiquitinated PHLPP and PHLPPL visualised by immunoblotting using an anti-ubiquitin antibody. In agreement with previous reports, both phosphatases were ubiquitinated in cells (Figure 2A, lanes 2 and 4). Importantly, overexpression of Usp12 deubiquitinated both PHLPP and PHLPPL (Figure 2A, lanes 1 and 3) and this elevated the steady-state levels of the enzymes, while overexpression of an enzymatically inactive Usp12_C48A_ mutant failed to elevate PHLPP and PHLPPL levels suggesting the importance of Usp12 enzymatic activity for phosphatase regulation (Figure 2B and Figure 2C). Similarly, when we silenced Usp12, or its interacting partners Uaf-1 and WDR20, levels of PHLPP and PHLPPL were decreased in LNCaP PC cells (Figure 2D).

As PHLPP and PHLPPL control the levels of active pAkt, we evaluated the effects of depleting components of the Usp12 complex on Akt status. Knockdown of Usp12, or its complex members Uaf-1 and WDR20, increased Akt phosphorylation without any change to the total Akt protein in PC cells (Figure 2E). Increased Akt activity was further confirmed by the increase in 4E-BP1 and ERK1/2 Akt target proteins phosphorylation levels (Figure 2E). Our results confirm that Usp12 controls Akt phosphorylation in PC cells by deubiquitinating and stabilising two Akt phosphatases PHLPP and PHLPPL.

### Usp12 regulates AR phosphorylation by controlling pAkt levels

**Figure 3:**
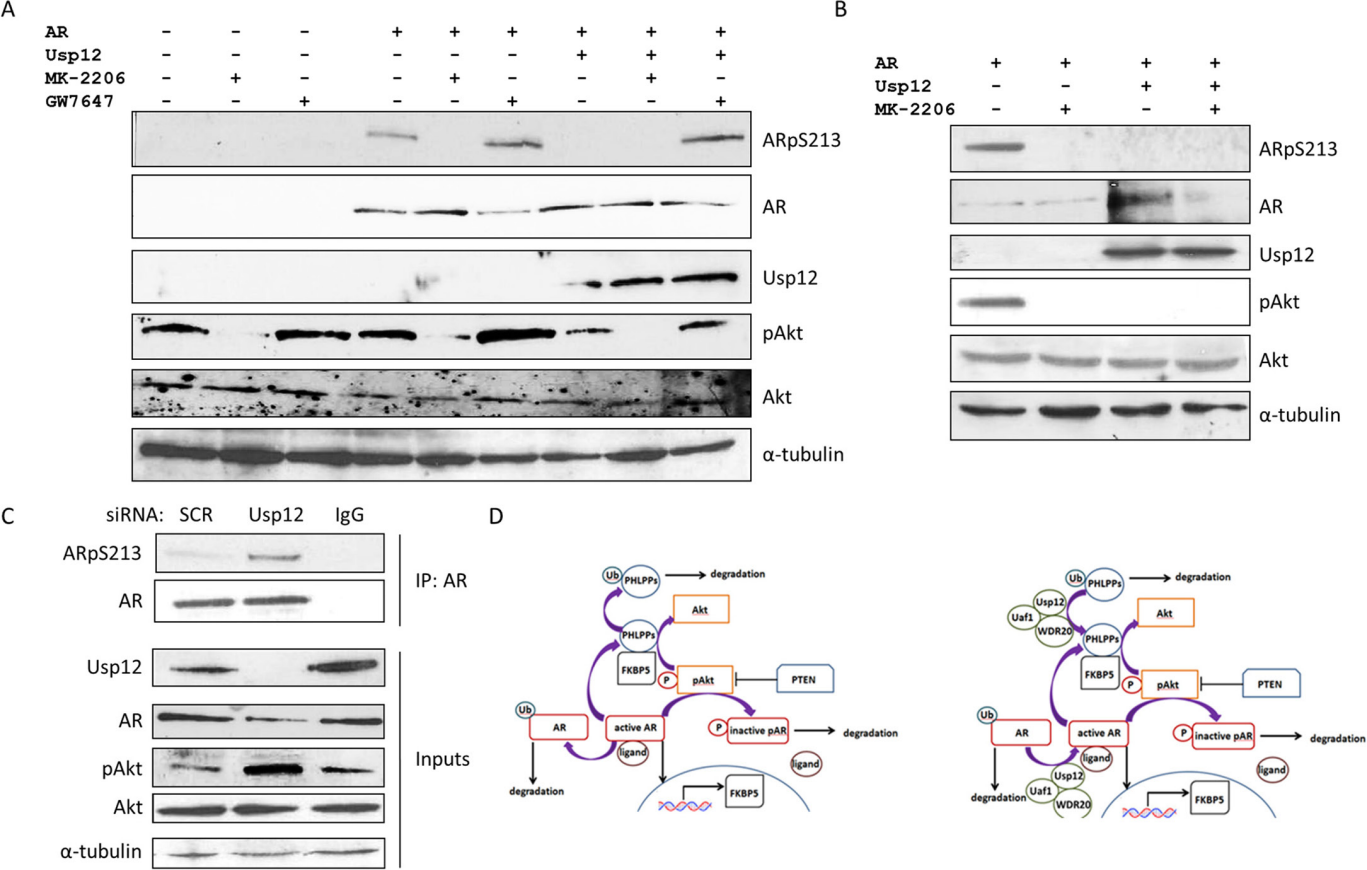
Usp12 controls the levels of AR Serine 213 phosphorylation by Akt **(A)** PC3 cells were transfected with AR and Usp12 plasmids as indicated. 48 hours post transfection cells were treated with 1μM MK-2206 or 1μM GW7647 for a further 24h. 72h post transfection cells were harvested and immunoblotted. **(B)** COS-7 cells were transfected with AR and Usp12 plasmids as indicated, 48h post transfection cells were treated with 1μM MK-2206 for a further 24h. 72h post transfection cells were harvested and immunoblotted. **(C)** LNCaP cells were treated with siRNA as indicated for 96h, following silencing cells were harvested and lysates were immunoprecipitated (IP) with AR antibody followed by immunoblotting. **(D)** Left panel, phosphorylated Akt (pAkt) phosphorylates AR resulting in ligand disassociation and increased AR degradation. This can be inhibited by PTEN. Additionally, PHLPP and PHLPPL chaperoned by FKBP5 dephosphorylate pAkt resulting in its inactivation (Akt). When Akt is inactive, AR remains bound to its ligand and translocates to the nucleus where it acts as a transcription factor for multiple androgen regulated genes, including FKBP5. This pathway is controlled by ubiquitination of both PHLPP and PHLPPL and AR leading to their proteasomal degradation. Right panel, predicted role of Usp12 as a regulator of the Akt pathway and AR interaction. Usp12 deubiquitinates AR rescuing it from proteosomal degradation. Additionally, Usp12 deubiquitinates PHLPP and PHLPPL resulting in decreased Akt activity, inhibition of AR Ser213 and Ser791 phosphorylation by Akt and enhanced AR stability.

Akt has been previously reported to phosphorylate AR at S213 and S791 [17]. We have recently shown that Usp12 has a direct effect on AR by deubiquitinating and stabilising the AR [13]. To assess if Usp12 additionally controls AR phosphorylation and activity by regulating pAkt levels, we overexpressed AR and Usp12 in the PC3 prostate cancer cell line and assessed the phosphorylation status of the receptor. AR S213 phosphorylation was reduced in response to Usp12 overexpression despite total AR protein levels remaining stable (Figure 3A, compare lanes 4 and 6). To confirm that changes in AR phosphorylation were caused by pAkt we analysed the levels of pS213AR after treatment with Akt inhibitor MK-2206. Akt inhibition abrogated S213 phosphorylation of AR and decreased the ability of Usp12 to stabilise AR protein levels (Figure 3A). Additionally, we assessed the levels of AR phosphorylation upon Usp12 inhibition, to this end we incubated PC3 cells in the presence of GW7647, a Usp1 and Usp12 inhibitor (unpublished data, Harle *et al*.). GW7647 caused an increase in pAkt levels and as a result increased AR S213 phosphorylation. We further confirmed this result by repeating this experiment in COS-7 cells where Usp12 had the same effect (Figure 3B). To assess if Usp12 regulates endogenous AR S213 modification, the receptor was immunoprecipitated from LNCaP cells depleted of Usp12 and phosphorylation assessed by immunoblotting. In agreement with the above result, knockdown of Usp12 in LNCaP cells increased both AR S213 phosphorylation and AR turnover (Figure 3C). Our data demonstrates that Usp12 increases the levels of PHLPP and PHLPPL that in turn decreases the active pAkt pool and inhibits AR S213 phosphorylation by Akt. This further explains our previous observation that Usp12 silencing has both cytotoxic and anti-proliferative effects on PCa cells [13]. We hypothesise that Usp12 controls the levels of AR both directly by deubiquitinating AR itself and indirectly by inhibiting Akt activity and AR S213 phosphorylation via stabilisation of PHLPP/L and as such Usp12 is a guardian of the interaction between the Akt and AR pathways (Figure 3D).

### S213 of AR is required for transcriptional co-activation by Usp12 and Uaf-1

**Figure 4:**
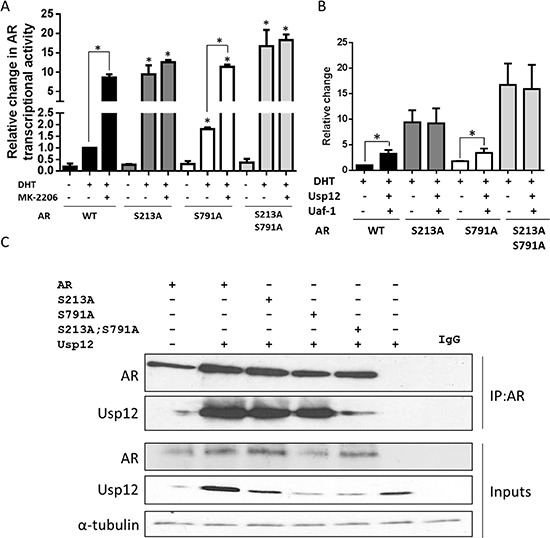
AR Ser213 is required for the upregulation of transcriptional activity by Usp12 and Uaf-1 **(A-B)** HEK293T cells were transfected with pARE3-luc, pCMV-b-gal, pFlag-AR **(A)** and pARE3-luc, pCMV-b-gal, pFlag-AR, pFlag-Usp12, and pFlag-Uaf-1 **(B)** as indicated and cultured for 72h in steroid depleted conditions followed by addition of 10 nM DHT for 24h where indicated. Results are represented as luciferase counts per second normalised to b-galactosidase activity. Data are a mean +/− SEM of three independent experiments normalised to WT AR alone, statistical significance was analysed with t-test. **(C)** COS-7 cells were transfected with WT, S213A, S791A and double S213A and S791A mutant AR alongside Usp12 as indicated. 72h post transfection cells were harvested and lysates were immunoprecipitated (IP) with AR antibody followed by immunoblotting.

AR S213/S791phosphorylation by Akt was reported to decrease AR-mediated transcription by inhibiting co-activator interaction and promoting receptor degradation [17]. To confirm these results, we created AR mutants deficient in these phosphorylation sites by substituting both serines with alanine. We then assessed their transcriptional activity in the absence and presence of androgen stimulation. In steroid-depleted conditions, only the double S213A S791A mutant had significantly increased transcriptional activity compared to wild type AR (Figure 4A). However, in the presence of DHT all mutants of AR had significantly increased transcriptional activity (Figure 4A). Interestingly, in agreement with previous reports, S213A substitution enhanced transcriptional activity of AR to a greater extent than that of S791A, with the most significant effect observed for the double mutant (Figure 4A). To ensure that this change in AR activity is a result of phosphorylation by Akt we treated cells with an Akt inhibitor. Akt inhibition increased transcriptional activity of wild type and both S213A and S791A mutants of AR but not of the double S213A; S791A mutant (Figure 4A). Our results confirm that phosphorylation of AR by Akt at both S213 and S791 decreases AR transcriptional activity.

We have recently reported that Usp12, in combination with Uaf-1, can increase the transcriptional activity of AR [13], and hence we wanted to analyse the importance of Akt phosphorylation sites in this process. Although both AR_S213A_ and AR_S213A/S791A_ demonstrated elevated transcriptional activity compared to wild-type, both were refractory to ectopically-expressed Usp12 and Uaf-1 (Figure 4B). To investigate if this was caused by the lack of interaction between AR and Usp12 in the absence of the specific serine residues, we overexpressed Usp12 alongside wild-type and mutant ARs followed by immunoprecipitation. Usp12 was capable of interacting with both AR_S213A_ and AR_S213A/S791A_ with comparable efficiency to wild-type confirming that these sites are not required for this interaction (Figure 4C). This data suggests that AR co-activation by Usp12 and Uaf-1 is largely driven through negating AR phosphorylation by Akt inactivation.

### Depleting Usp12 sensitises PC cells to Akt inhibition

**Figure 5:**
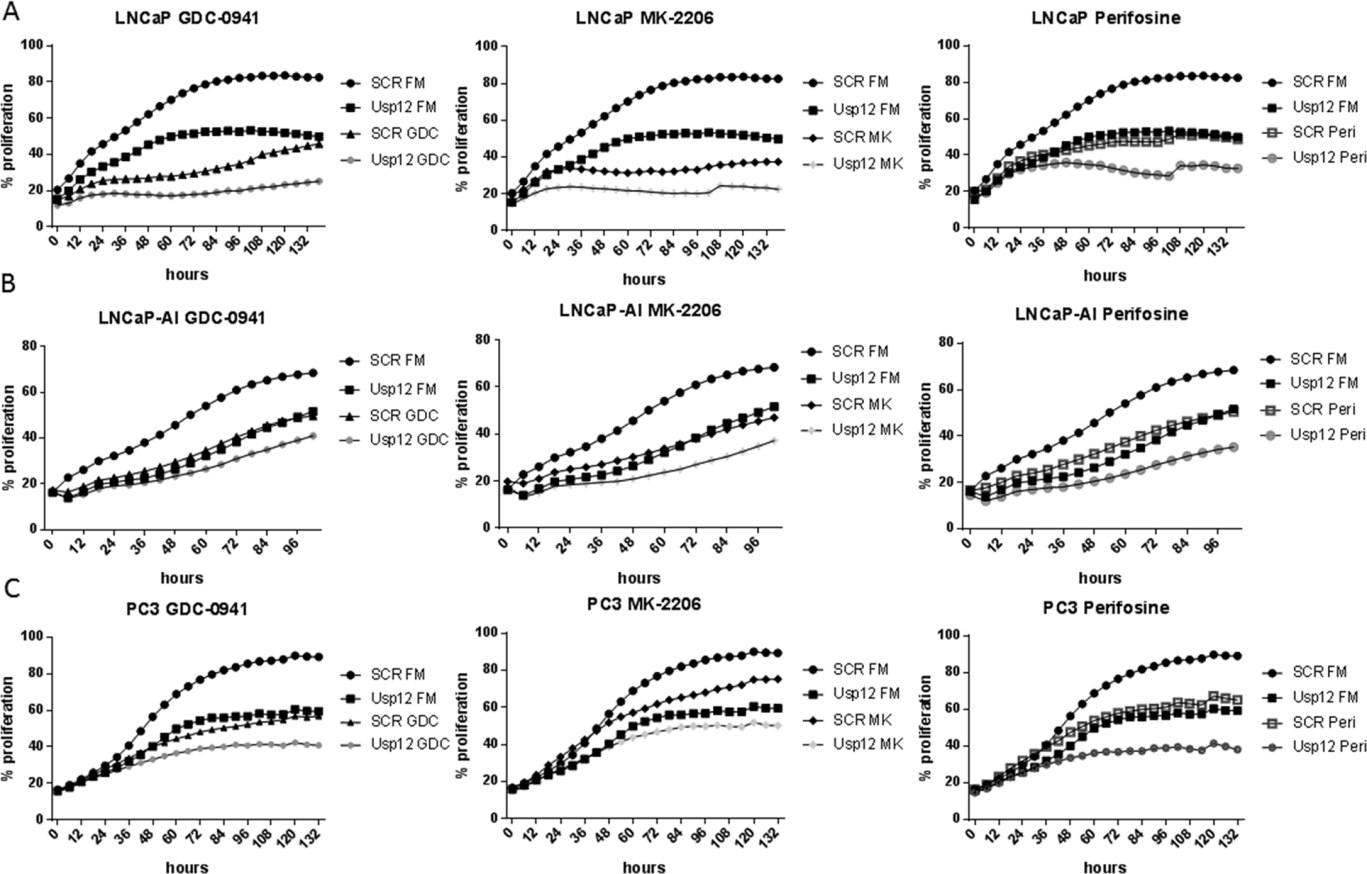
Usp12 depletion sensitises PC cells to Akt inhibition irrespectively of their castration sensitivity and AR status indicated by decreased cellular occupation of the wells **(A-C)** LNCaP, LNCaP-AI and PC3 cells respectively were treated with siRNA and compounds as indicated. Cells were grown in their respective full media (FM); LNCaP and PC3 cells were grown in steroid containing media and LNCaP-AI were grown in steroid depleted media. Cellular occupation of the wells was measured every 4h using the IncuCyte system. Data are a mean of three independent experiments.

**Figure 6:**
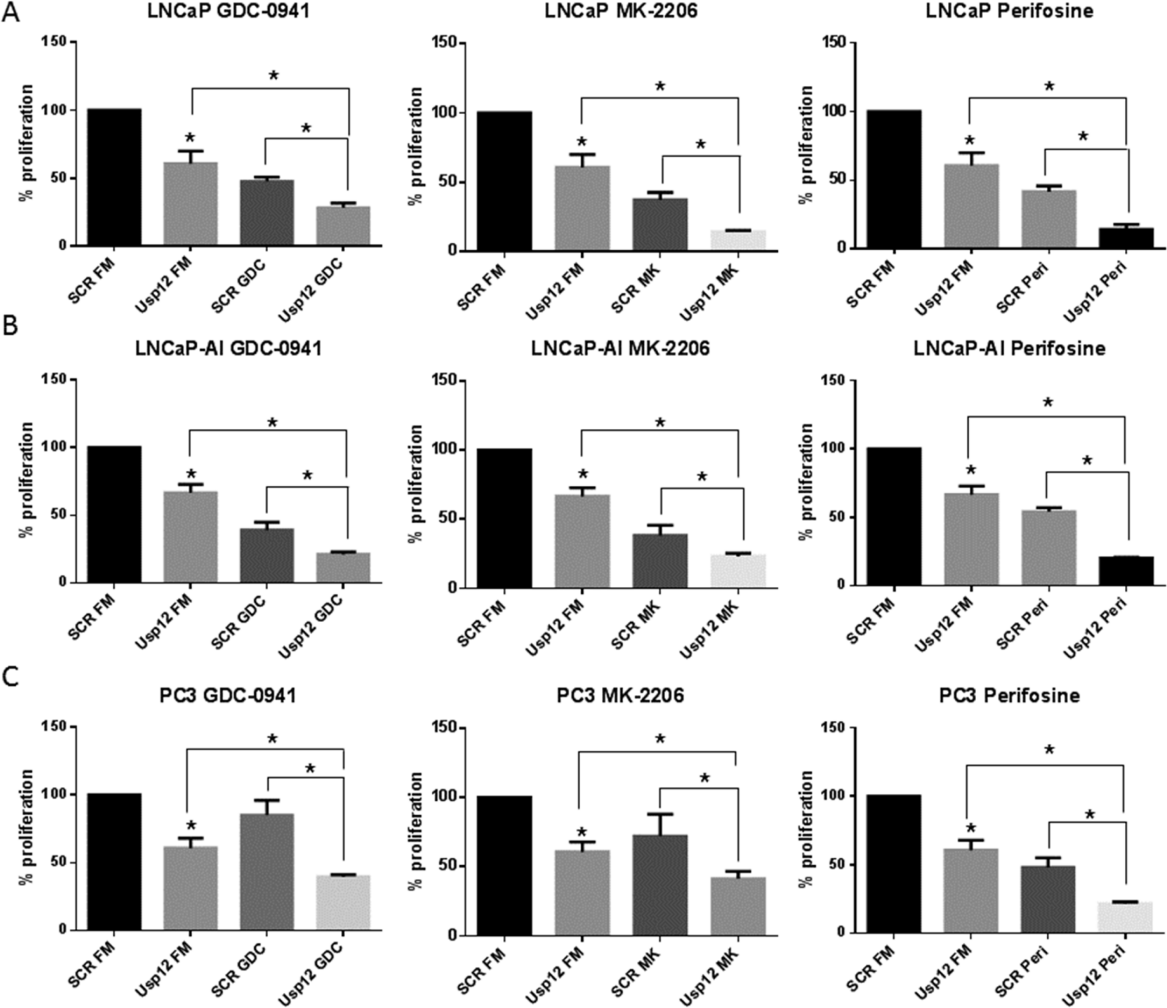
Usp12 depletion is an independent sensitiser to therapies aimed at Akt **(A-C)** LNCaP, LNCaP-AI and PC3 cells respectively were treated with siRNA and compounds as indicated. Cells were grown in their respective full media (FM); LNCaP and PC3 cells were grown in steroid containing media and LNCaP-AI were grown in steroid depleted media. Cellular proliferation was measured after 96h by cell counting. Data are a mean +/− SEM of three independent experiments. Statistical significance was analysed using t-test.

Our data confirms that Usp12 controls the levels of pAkt in PC cells by deubiquitinating and stabilising two Akt phosphatases PHLPP and PHLPPL. Depleting Usp12 results in increased pAkt and as such predisposes Akt to be a major driver of cellular proliferation under those conditions. To assess the impact of Usp12 depletion on Akt inhibition we used three different compounds, GDC-0941 which is a PI3K inhibitor acting upstream of Akt and two direct Akt inhibitors MK-2206 and Perifosine. We analysed cellular proliferation in LNCaP (castration sensitive), LNCaP-AI (castration resistant) and PC3 (AR negative) PC cells using two separate assays. We report that depleting Usp12 significantly sensitised PC cells to Akt inhibition irrespectively of their castration sensitivity or AR status (Figure 5A-C and 6A-C).

## DISCUSSION

Regulation of AR protein levels and activity by post-translational modifications is known to regulate both normal and malignant prostate cells. Alterations in this control system have been reported to affect disease outcome and survival prognosis [30]. This is demonstrated by the continued importance of the AR in CRPC where it still remains the main focus of therapeutic strategies [2]. Therapies aimed at the AR invariably fail as a result of AR becoming promiscuous through mutations, acquiring the ability to become activated by a variety of steroid based ligands and anti-androgens, and AR amplification [2]. Therefore, a possible strategy for new CRPC therapeutics is to focus on upstream co-regulators of the AR as these directly control transcriptional potency of the receptor. PC development and progression is affected by Akt signalling which promotes cellular growth and proliferation, and acts in a feedback loop with the AR [14]. Akt activity is also regulated by post-translational modifications with tight regulation being imposed by phosphatases including PTEN, PHLPP and PHLPPL [31].

Usp12 was previously predicted to interact with PHLPPs using proteomics tools [27], as a result the interplay between Usp12 and PHLPPs was investigated. We demonstrated that Usp12, and two Usp12-interacting proteins, Uaf-1 and WDR20 [28], [29], form a complex with PHLPP and PHLPPL. Further analysis using denaturing immunoprecipitation experiments revealed that Usp12 can also deubiquitinate and stabilise both PHLPP and PHLPPL. This is the first report detailing the regulation of PHLPPL by deubiquitination, as PHLPPL dephosphorylates distinct isoforms of Akt than PHLPP it is crucial to understand the post-translational regulation of this phosphatase. Additionally, we found that in the presence of Usp12, AR S213 phosphorylation, an Akt target site, was decreased suggesting that Akt activity was reduced. This was confirmed in PC cells where Usp12 depletion caused an increase in both pAkt and phosphorylated AR S213 levels.

We demonstrate that Usp12 plays a crucial role in PC by firstly, directly deubiquitinating the AR and rescuing it from proteosomal degradation resulting in increased AR protein and activity [13]. Secondly, Usp12 deubiquitinates and stabilises PHLPP and PHLPPL proteins which enhances dephosphorylation and deactivation of Akt. Consequently, pAkt levels are decreased inhibiting subsequent S213AR phosphorylation and thereby increasing the pool of transcriptionally active AR which is the main driver of PC tumourigenesis. Our results indicate that direct activity of Usp12 resulting in AR deubiquitination might be more important to maintain AR protein levels [13] where indirect action via Akt controls AR transcriptional activity (Figure 4B). This supports previous reports that AR S213 phosphorylation by Akt causes dissociation of AR from its co-factors resulting in decreased transcriptional activity [16]. We hypothesise that Usp12 might be a crucial regulator of the balance between cell survival and apoptosis by acting as a master regulator of the known feedback loop between AR and the PI3K/Akt pathway.

It is not uncommon for the same DUB to regulate AR in both a direct and indirect manner. Usp10, another DUB reported to target AR, was also shown to enhance AR activity by combining both direct and indirect activity. Usp10 can bind to AR and positively regulate its transcriptional activity [12] however, it also regulates AR indirectly via deubiquitination of H2A.Z causing the same effect [32]. Our study adds to this model highlighting the complexity in regulation of cellular pathways.

Similarly to Akt, PIM-1S was demonstrated to phosphorylate AR at S213 resulting in AR degradation and inhibition of its transcriptional activity [19]. PIM-1S works within a feedback loop with PIM-1L which has an opposite effect [19]. Further investigation of the relationship between Usp12 and PIM-1S and PIM-1L might uncover an additional layer of AR regulation by Usp12. Our results suggest that Usp12 might be a valid drug target. We found that depleting Usp12 protein resulted in the sensitisation of prostate cancer cells to Akt inhibition irrespectively of their androgen sensitivity and AR status. Interestingly, inhibition of Usp1, a close homologue of Usp12 has been shown to have promising results in lung cancer systems which was achieved by inhibiting the Usp1-Uaf-1 complex [33], [34]. The same approach could potentially be employed to inhibit Usp12.

## MATERIALS AND METHODS

### Antibodies, plasmids and reagents

The following antibodies were used during this project anti-Flag, anti-Uaf-1, anti-Usp12, anti-a-tubulin and anti-PHLPP provided by Sigma. Anti-AR (N20 clone), anti-HA (Y11 clone), anti-WDR20, anti-Ubiquitin, anti-ERK2, anti-pERK1/2, anti-Akt and anti-pAkt provided by Santa Cruz. Anti-PHLPPL (Bethyl), anti-p21 (Calbiochem), anti-p4EBP1, anti-4EBP1 (Cell Signalling) and anti-pS213 AR (Abcam). Plasmids used were pARE3-Luc, pCMV-b-gal, pFlag-His-AR [35]. pFlag-His-AR and its S213A, S791A and double S213A;S791A mutants were generated in house by *in vitro* mutagenesis (Quickchange, Stratagene), pFlag-Usp12 wild type and C48A mutant [13], pHA-Ubiquitin and pHA-Flag-WDR20 and pFlag-Uaf-1 [36]; [28] which were kind gifts from Professor Alan D'Andrea (Dana-Farber Cancer Institute, Boston), pHA-PHLPP [37] and pHA-PHLPPL [21] purchased from Addgene. For the assessment of Akt inhibition with and without Usp12 silencing we used Akt inhibitors MK-2206 dihydrochloride and Perifosine from Addooq Bioscience and PI3K inhibitor GDC-0941 from Selleckchem.

### Cell culture, transfections and reporter assays

LNCaP, HEK293T, PC3 and COS-7 cells were obtained from American Type Culture Collection (Manassas, USA). Cells were cultured in RPMI 1640 media with 2 mM L-glutamine (Invitrogen) supplemented with 10% (v/v) foetal calf serum (FCS) at 37°C in 5% CO_2_. LNCaP-AI variant cell line was derived in-house by culturing LNCaP cells in steroid-depleted media to allow for the development of androgen independence [38]. Transfections were performed using TransIT-LT1 reagent (Mirus Bio) following the manufacturer's instructions.

For luciferase assays, cells were transfected with 50 ng pARE3-luc, 10 ng pCMV-b-gal and 10 ng of pFlag-His-AR, pFlag-Usp12 and pFlag-Uaf-1 as indicated. All reactions were balanced with pCMV empty vector. Cells were cultured under steroid depleted conditions for 72h followed by supplementation with 10nM dihydrotestosterone (DHT) for additional 24h. Cells were lysed and incubated in 1x Promega luciferase assay reagent according to the manufacturer's instruction and luciferase counts per second were established and normalised to b-galactosidase activity. Results were normalised to AR expression alone in steroid depleted conditions.

### siRNA gene silencing and gene expression analysis

Usp12 targeting siRNA sequence was CAGAUCUCUUCCAUAGCAU[dTdT], WDR20 was silenced with siRNA CGAGAAAGAUCACAA GCGA[dTdT] and Uaf-1 with CAAAUUGGUUC UCAGUAGA[dTdT]. Routinely, we achieved >60%, >65% and >80% knockdown for Uaf-1, WDR20 and Usp12, respectively in qPCR validation (data not shown). LNCaP, LNCaP-AI and PC3 cells were reverse transfected with siRNA using RNAiMax (Invitrogen) according to manufacturer's instructions and incubated in culture media for 96h prior to cell lysis and analysis by Western blotting as described previously [13].

### Proliferation analysis

For proliferation analysis, cells were transfected with siRNA and treated either with DMSO (controls) or with PI3K inhibitor GDC-0941 (0.5μM) or Akt inhibitors MK-2206 (1μM) or Perifosine (5μM). IncuCyte measurements of cellular occupation of the wells were taken every 4 hours and additionally in a separate set of experiments cell numbers were counted at 96h to assess cellular proliferation.

### Immunoprecipitations

Cells were seeded at 5×10^5^ cells per 90 mm dish and transfected with 1μg of each plasmid as indicated, incubated for 72h and lysed directly into lysis buffer (50 mM Tris pH 7.5, 150 mM NaCl, 0.2 mM Na_3_VO_4_, 1% NP-40, 1 mM PMSF, 1 mM DTT and 1x protease inhibitors (Roche)). Lysates were incubated with 1 μg of antibodies as indicated for 16h at 4°C, antibodies were pulled down using Protein G Sepharose beads. For denaturing IPs, cells were subjected to 20 μM of MG-132 proteosomal inhibitor treatment for the final 16h followed by collection into lysis buffer with an addition of 2% SDS and denatured at 100°C for 10 minutes [13]. Following denaturation, samples were diluted 10x in lysis buffer without SDS and processed as in native IP. Immunoprecipitates were analysed using Western blotting.

## References

[1] Culig Z (2003). Role of the androgen receptor axis in prostate cancer. Urology.

[2] Feldman BJ, Feldman D (2001). The development of androgen-independent prostate cancer. Nature Reviews Cancer.

[3] Ryan CJ, Smith A, Lal P, Satagopan J, Reuter V, Scardino P, Gerald W, Scher HI (2006). Persistent prostate-specific antigen expression after neoadjuvant androgen depletion: An early predictor of relapse or incomplete androgen suppression. Urology.

[4] Gaughan L, Logan IR, Neal DE, Robson CN (2005). Regulation of androgen receptor and histone deacetylase 1 by Mdm2-mediated ubiquitylation. Nucleic Acids Rese-arch.

[5] Lin HK, Wang L, Hu YC, Altuwaijri S, Chang C (2002). Phosphorylation-dependent ubiquitylation and degradation of androgen receptor by Akt require Mdm2 E3 ligase. EMBO J.

[6] He B, Bai S, Hnat AT, Kalman RI, Minges JT, Patterson C, Wilson EM (2004). An androgen receptor NH2-terminal conserved motif interacts with the COOH terminus of the Hsp70-interacting protein (CHIP). The Journal of Biological Chemistry.

[7] Cardozo CP, Michaud C, Ost MC, Fliss AE, Yang E, Patterson C, Hall SJ, Caplan AJ (2003). *C*-terminal Hsp- interacting protein slows androgen receptor synthesis and reduces its rate of degradation. Archives of Biochemistry and Biophysics.

[8] Xu K, Shimelis H, Linn DE, Jiang R, Yang X, Sun F, Guo Z, Chen H, Li W, Kong X, Melamed J, Fang S, Xiao Z, Veenstra TD, Qiu Y (2009). Regulation of androgen receptor transcriptional activity and specificity by RNF6-induced ubiquitination. Cancer Cell.

[9] Li H, Xu LL, Masuda K, Raymundo E, McLeod DG, Dobi A, Srivastava S (2008). A feedback loop between the androgen receptor and a NEDD4-binding protein, PMEPA1, in prostate cancer cells. The Journal of Biological Chemistry.

[10] Shenoy SK, Xiao K, Venkataramanan V, Snyder PM, Freedman NJ, Weissman AM (2008). Nedd4 mediates agonist-dependent ubiquitination, lysosomal targeting, and degradation of the beta2-adrenergic receptor. The Journal of Biological Chemistry.

[11] Dirac AM, Bernards R (2010). The deubiquitinating enzyme USP26 is a regulator of androgen receptor signaling. Molecular Cancer Research.

[12] Faus H, Meyer HA, Huber M, Bahr I, Haendler B (2005). The ubiquitin-specific protease USP10 modulates androgen receptor function. Molecular and Cellular Endocrinology.

[13] Burska UL, Harle VJ, Coffey K, Darby S, Ramsey H, O'Neill D, Logan IR, Gaughan L, Robson CN (2013). Deubiquitinating enzyme Usp12 is a novel co-activator of the Androgen Receptor. The Journal of Biological Chemistry.

[14] Sarker D, Reid AH, Yap TA, de Bono JS (2009). Targeting the PI3K/AKT pathway for the treatment of prostate cancer. Clinical Cancer Research.

[15] Assinder SJ, Dong Q, Kovacevic Z, Richardson DR (2009). The TGF-beta, PI3K/Akt and PTEN pathways: established and proposed biochemical integration in prostate cancer. The Biochemical Journal.

[16] Lin HK, Yeh S, Kang HY, Chang C (2001). Akt suppresses androgen-induced apoptosis by phosphorylating and inhibiting androgen receptor. Proceedings of the National Academy of Sciences of the United States of America.

[17] Palazzolo I, Burnett BG, Young JE, Brenne PL, La Spada AR, Fischbeck KH, Howell BW, Pennuto M (2007). Akt blocks ligand binding and protects against expanded polyglutamine androgen receptor toxicity. Human Molecular Genetics.

[18] Wen Y, Hu MC, Makino K, Spohn B, Bartholomeusz G, Yan DH, Hung MC (2000). HER-2/neu promotes androgen-independent survival and growth of prostate cancer cells through the Akt pathway. Cancer Resea-rch.

[19] Ha S, Iqbal NJ, Mita P, Ruoff R, Gerald WL, Lepor H, Taneja SS, Lee P, Melamed J, Garabedian MJ, Logan SK (2013). Phosphorylation of the androgen receptor by PIM1 in hormone refractory prostate cancer. Oncogene.

[20] McCall P, Gemmell LK, Mukherjee R, Bartlett JMS, Edwards J (2008). Phosphorylation of the androgen receptor is associated with reduced survival in hormone-refractory prostate cancer patients. Br J Cancer.

[21] Brognard J, Sierecki E, Gao T, Newton AC (2007). PHLPP and a second isoform, PHLPP2, differentially attenuate the amplitude of Akt signaling by regulating distinct Akt isoforms. Molecular Cell.

[22] O'Neill AK, Niederst MJ, Newton AC (2013). Suppression of survival signalling pathways by the phosphatase PHLPP. FEBS Journal.

[23] Li X, Liu J, Gao T (2009). beta-TrCP-mediated ubiquitination and degradation of PHLPP1 are negatively regulated by Akt. Molecular Cell Biology.

[24] Li X, Stevens PD, Yang H, Gulhati P, Wang W, Evers BM, Gao T (2013). The deubiquitination enzyme USP46 functions as a tumor suppressor by controlling PHLPP-dependent attenuation of Akt signaling in colon cancer. Oncogene.

[25] Zhiqiang Z, Qinghui Y, Yongqiang Z, Jian Z, Xin Z, Haiying M, Yuepeng G (2012). USP1 regulates AKT phosphorylation by modulating the stability of PHLPP1 in lung cancer cells. Journal of Cancer Research and Clinical Oncology.

[26] Gangula NR, Maddika S (2013). WD Repeat Protein WDR48 in Complex with Deubiquitinase USP12 Suppresses Akt-dependent Cell Survival Signaling by Stabilizing PH Domain Leucine-rich Repeat Protein Phosphatase 1 (PHLPP1). The Journal of Biological Chemistry.

[27] Sowa ME, Bennett EJ, Gygi SP, Harper JW (2009). Defining the Human Deubiquitinating Enzyme Interaction Landscape. Cell.

[28] Kee Y, Yang K, Cohn MA, Haas W, Gygi SP, D'Andrea AD (2010). WDR20 regulates activity of the USP12 x UAF1 deubiquitinating enzyme complex. The Journal of Biological Chemistry.

[29] Faesen Alex C, Luna-Vargas Mark PA, Geurink Paul P, Clerici M, Merkx R, van Dijk Willem J, Hameed Dharjath S, El Oualid F, Ovaa H, Sixma Titia K (2011). The differential modulation of USP activity by internal regulatory domains, interactors and eight ubiquitin chain types. Chemistry and Biology.

[30] Coffey K, Robson CN (2012). Regulation of the androgen receptor by post-translational modifications. The Journal of Endocrinology.

[31] Molina JR, Agarwal NK, Morales FC, Hayashi Y, Aldape KD, Cote G, Georgescu MM (2012). PTEN, NHERF1 and PHLPP form a tumor suppressor network that is disabled in glioblastoma. Oncogene.

[32] Draker R, Sarcinella E, Cheung P (2011). USP10 deubiquitylates the histone variant H2A.Z and both are required for androgen receptor-mediated gene activation. Nucleic Acids Research.

[33] Chen J, Dexheimer TS, Ai Y, Liang Q, Villamil MA, Inglese J, Maloney DJ, Jadhav A, Simeonov A, Zhuang Z (2011). Selective and cell-active inhibitors of the USP1/ UAF1 deubiquitinase complex reverse cisplatin resistance in non-small cell lung cancer cells. Chemistry and Biology.

[34] Liang Q, Dexheimer TS, Zhang P, Rosenthal AS, Villamil MA, You C, Zhang Q, Chen J, Ott CA, Sun H, Luci DK, Yuan B, Simeonov A, Jadhav A, Xiao H, Wang Y (2014). A selective USP1-UAF1 inhibitor links deubiquitination to DNA damage responses. Nature Chemical Biology.

[35] Brady ME, Ozanne DM, Gaughan L, Waite I, Cook S, Neal DE, Robson CN (1999). Tip60 is a nuclear hormone receptor coactivator. The Journal of Biological Chemistry.

[36] Cohn MA, Kee Y, Haas W, Gygi SP, D'Andrea AD (2009). UAF1 is a subunit of multiple deubiquitinating enzyme complexes. The Journal of Biological Chemistry.

[37] Gao T, Furnari F, Newton AC (2005). PHLPP: a phosphatase that directly dephosphorylates Akt, promotes apoptosis, and suppresses tumor growth. Molecular Cell.

[38] Lu S, Tsai SY, Tsai MJ (1999). Molecular mechanisms of androgen-independent growth of human prostate cancer LNCaP-AI cells. Endocrinology.

